# Editorial: Targeting secondary brain damage following intracerebral hemorrhage: from bench to bedside, volume II

**DOI:** 10.3389/fnins.2024.1413483

**Published:** 2024-05-02

**Authors:** Cheng Gao

**Affiliations:** Department of Neurosurgery, The First Affiliated Hospital of Harbin Medical University, Harbin, China

**Keywords:** intracerebral hemorrhage, secondary brain injury, oxidative stress, hypothermia, thrombin

Intracerebral hemorrhage (ICH), the most common subtype of hemorrhagic stroke which accounts for 10%−15% of all stroke cases, often causes severe mortality or disability (Tsao et al., 2022). Classically, injury from ICH is separated into primary brain injury (PBI), the initial damage from the hemorrhage itself, such as the hematoma, leading to increased intracranial pressure and subsequent structural disruption, and secondary brain injury (SBI), the damage related to downstream pathways activated in the presence of intraparenchymal blood, including inflammation, oxidative stress, blood-brain barrier breakdown, cytotoxicity, and neuronal death (Magid-Bernstein et al., 2022). Currently, the treatments for PBI, targeting hematoma expansion and mass effect, fail to demonstrate significant benefits (Cordonnier et al., 2018). Emerging evidence has indicated that the duration of SBI is longer and the damage is often more serious than PBI induced by the direct mechanical effects of the hematoma, however, there exists no evidence-based medical treatment for SBI followed ICH (Loan et al., 2022). Therefore, there is an urgent need to clarify the mechanisms of SBI and seek effective treatment strategies to increase the survival rate and improve the quality of life for survivors. This Research Topic on “*Targeting secondary brain damage following intracerebral hemorrhage: from bench to bedside Volume II*” comprises four articles that add some measures to prevent and treat SBI after ICH in preclinical ICH models and clinical trials based on Volume I.

Oxidative stress is implicated in the genesis and development of SBI, causing oxidative damage of biological macromolecules, BBB disruption, inflammation, neuronal cell death (Chen et al., 2022). The major contributor to oxidative stress is the release of hemoglobin and its breakdown products from erythrocytes (Magid-Bernstein et al., 2022). Yan et al. emphasized the importance of oxidative stress in SBI after ICH and demonstrated that cannabidiolz (CBD) could inhibit oxidative stress generation from mitochondria dysfunction, excitotoxicity, iron toxicity and inflammatory cells. CBD can not only directly scavenge radicals but also exert antioxidant effects through specific receptor-mediated pathways, such as endocannabinoid receptors CB1 and CB2.

Fever is common in the first 72 h following ICH, occurring in 30% to 45% of patients (Magid-Bernstein et al., 2022), the duration of fever within 72 h after admission to the hospital was correlated with clinical outcomes, which provided a theoretical basis for active treatment of fever to maintain normal body temperature in patients with ICH (Diringer et al., 2004). Cadena et al. introduced an innovative therapeutic strategy for SBI from ICH, therapeutic hypothermia. Hypothermia has gained increasing interest due to its potential to minimize systemic complications and improve outcomes, which hints that therapeutic hypothermia may be a promising treatment for SBI from ICH.

Tao et al. summarized the two side effects of thrombin in SBI and the promising application potential of it. Thrombin-induced brain injury is concentration-dependent, with high concentrations causing BBB injury, brain edema, and neuronal apoptosis, and low concentrations promoting neuronal growth and branching, improving neuronal viability, and preventing excitotoxic injury. The administration of thrombin inhibitors, such as hirudin and argatroban, can improve thrombin-induced injury post-ICH by directly suppressing the activity of thrombin, which offers a novel way for future SBI treatment after ICH. Administration of thrombin inhibitors following ICH has been shown to effectively mitigate neuroinflammation and brain edema, thereby enhancing prognosis. However, the comprehensive suppression of thrombin activity would yield adverse consequences due to the demonstrated neuroprotective and angiogenic properties associated with low levels of thrombin. Consequently, excessive utilization of thrombin inhibitors may exacerbate secondary injury and impede the prospects of long-term recuperation. Therefore, how to administer thrombin inhibitor reasonably is the key to its clinical application.

ICH resultant complications are essential factors in the prognosis of ICH patients, such as ischemic stroke and ischemic cardiovascular disease, which account for nearly 15% of deaths after ICH (Li and Murthy, 2022). Besides, the crosstalk between other diseases and ICH is also worthy of exploring, Song et al. analyzed the association between ovarian cancer and stroke, and the results indicated that bevacizumab in combination with chemotherapy may not increase the risk of stroke, including intracerebral hemorrhage, which remind us that attention should be paid not only to ICH itself and its complications but also to the association of other diseases with ICH, and broaden the horizon for preventing ICH.

To summarize, articles in Volume II of this Research Topic supplement some exact treatment strategies and clinical issues of SBI for Volume I. Notably, this set of articles provides the crosstalk between bevacizumab in combination with chemotherapy of ovarian cancer patients and ICH, the antioxidant effects of cannabidiol on ICH, the promising application of therapeutic hypothermia and the potential molecular mechanisms of thrombin-mediated effects and application prospect of thrombin treatment. Overall, further research is needed to find effective clinical treatment for SBI, which can benefit ICH patients more with the combination of surgical intervention in the acute phase targeting PBI.

## Author contributions

CG: Writing – original draft, Writing – review & editing.
